# The beneficial effects of non-specific synaptic plasticity for pattern recognition in auto-associative memory

**DOI:** 10.1186/1471-2202-12-S1-P316

**Published:** 2011-07-18

**Authors:** Lee Calcraft, Reinoud Maex, Neil Davey, Volker Steuber

**Affiliations:** 1 School of Computer Science, University of Hertfordshire, Hatfield, Herts, AL10 9AB, UK

## 

Most learning theories for artificial neural networks and real neural systems assume weight changes that are specific to activated synapses. However, experimental evidence suggests that different forms of synaptic plasticity such as long-term potentiation in hippocampal CA1 pyramidal cells [1] and long-term depression in cerebellar Purkinje cells [2][3] are not completely synapse specific, and also affect inactive synapses in the neighborhood of the activated ones. The functional role of this non-specific synaptic plasticity (NSSP) is not clear.

We explore in simulation the effect of NSSP during the training cycle of an associative memory, finding that when the presence of NSSP causes weight changes of nearby synapses to influence each other during training, the network becomes more resilient to performance degradation when patterns are shifted from their original training positions during attempted recall. Indeed, recall of shifted patterns was found to be better in networks with NSSP than in networks without.

A fully connected one-dimensional associative memory using perceptron training rules was trained on a set of unbiased patterns in which *on* or *off* nodes appear in small clusters*[4]. The network was then tested on noisy versions of the original patterns, and a measure of network performance was taken. The test patterns were then shifted by one position relative to the training patterns before random noise was added, and performance measures were again taken. This was repeated with progressively greater shifts in the test patterns. As expected, it was found that the greater the shift in the test pattern with respect to the original, the more poorly the network performed.

However, when we altered the training rule to incorporate NSSP, so that each time a weight was adjusted during training, weights on nearby synapses** were also changed, unexpected results were seen. It was found that although in cases where the test pattern set had not been shifted, the network with NSSP performed less well than the network without it, in cases where the test pattern set *had* been shifted the network with NSSP performed better than the network without.

This suggests that the performance of associative memories required to recognize patterns that are not precisely aligned with patterns that the network has trained on could be improved when synapses are allowed to influence other synapses in their neighborhood during training, which indicates a potential functional role for NSSP in the brain.

*These tests were carried out with networks of both 100 and 500 nodes, and with patterns with a mean cluster length of 5 units.

**Varying amounts of leakage were used in the training routines, affecting 1, 2 ,3 ,4 or 5 of the synapses either side of the target synapse. In these tests we assume the 'ideal' case in which axons emanating from physically adjacent nodes in the network terminate at synapses which are also physically adjacent to one another.
